# Association between Retinal Thickness Variability and Visual Acuity Outcome during Maintenance Therapy Using Intravitreal Anti-Vascular Endothelial Growth Factor Agents for Neovascular Age-Related Macular Degeneration

**DOI:** 10.3390/jpm11101024

**Published:** 2021-10-14

**Authors:** Timothy Y. Y. Lai, Ricky Y. K. Lai

**Affiliations:** 1 Department of Ophthalmology & Visual Sciences, The Chinese University of Hong Kong, Central Ave, Shatin, New Territories, Hong Kong, China; 2 2010 Retina & Macula Centre, Tsim Sha Tsui, Kowloon, Hong Kong, China; lai_r@yahoo.com

**Keywords:** neovascular age-related macular degeneration, anti-VEGF therapy, optical coherence tomography, retinal thickness, visual acuity, variability

## Abstract

Previous studies based on clinical trial data have demonstrated that greater fluctuations in retinal thickness during the course of intravitreal anti-vascular endothelial growth factor (anti-VEGF) therapy for neovascular age-related macular degeneration (nAMD) is associated with poorer visual acuity outcomes. However, it was unclear whether similar findings would be observed in real-world clinical settings. This study aimed to evaluate the association between retinal thickness variability and visual outcomes in eyes receiving anti-VEGF therapy for nAMD using pro re nata treatment regimen. A total of 64 eyes which received intravitreal anti-VEGF therapy (bevacizumab, ranibizumab or aflibercept) for the treatment of nAMD were evaluated. Variability in spectral-domain optical coherence tomography (OCT) central subfield thickness (CST) was calculated from the standard deviation (SD) values of all follow-up visits after three loading doses from month 3 to month 24. Eyes were divided into quartiles based on the OCT CST variability values and the mean best-corrected visual acuity values at 2 years were compared. At baseline, the mean ± SD logMAR visual acuity and CST were 0.59 ± 0.39 and 364 ± 113 µm, respectively. A significant correlation was found between CST variability and visual acuity at 2 years (Spearman’s ρ = 0.54, *p* < 0.0001), indicating that eyes with lower CST variability had better visual acuity at 2 years. Eyes with the least CST variability were associated with the highest mean visual acuity improvement at 2 years (quartile 1: +9.7 letters, quartile 2: +1.1 letters, quartile 3: −2.5 letters, quartile 4: −9.5 letters; *p* = 0.018). No significant difference in the number of anti-VEGF injections was found between the four CST variability quartile groups (*p* = 0.21). These findings showed that eyes undergoing anti-VEGF therapy for nAMD with more stable OCT CST variability during the follow-up period were associated with better visual outcomes. Clinicians should consider adopting treatment strategies to reduce CST variability during the treatment course for nAMD.

## 1. Introduction

Neovascular age-related macular degeneration (nAMD) is one of the most common causes of visual impairment in adults aged 50 years or older, especially in high-income regions [1]. Intravitreal anti-vascular endothelial growth factor (anti-VEGF) therapy is currently the standard-of-care treatment for patients with nAMD, and anti-VEGF therapy has been shown to be highly effective in reducing blindness due to nAMD [2,3,4,5]. Various treatment regimens using anti-VEGF therapy for nAMD have been adopted; these include proactive strategies such as fixed dosing and treat-and-extend, as well as more reactive approaches such as pro re nata (PRN) dosing and observe-and-plan [6,7,8,9,10]. Several studies and treatment guidelines have suggested that proactive treatment strategies such as fixed dosing and treat-and-extend dosing strategies might result in better visual acuity outcomes than PRN dosing when treating patients with nAMD [8,9,10,11,12]. However, a recent systematic review has demonstrated that after adjusting for the number of intravitreal anti-VEGF injections, neither the treatment dosing regimen adopted, nor the anti-VEGF agent used, were significant predictors for visual acuity changes [13]. Nonetheless, other factors such as age, anatomical status of the retina, including the presence or absence of subretinal and/or intraretinal fluids, optical coherence tomography (OCT) central macular thickness, and macular morphology have been implicated as important prognostic factors in determining visual outcomes [14,15,16].

Post hoc analyses of data from several clinical trials have evaluated the potential influence of OCT retinal thickness variation during the course of anti-VEGF therapy on the visual acuity outcomes of patients with nAMD [17,18]. The findings demonstrated that eyes which had greater fluctuation in the OCT retinal thickness during the course of anti-VEGF therapy had poorer visual acuity outcomes than eyes which had less fluctuation. However, most of these results were based on phase 3 clinical trial data with protocol-driven treatment, and it is unclear whether similar findings will be observed in the real-world clinical settings. The aim of our study was to evaluate the association between OCT retinal thickness fluctuation and visual acuity outcomes in patients receiving intravitreal anti-VEGF therapy in the real-world clinic setting using a personalized PRN dosing regimen.

## 2. Materials and Methods

### 2.1. Study Design

This was a retrospective study of consecutive eyes which received intravitreal anti-VEGF therapy from October 2012 to March 2018 for nAMD in the 2010 Retina and Macula Centre, Hong Kong. The inclusion criteria included the following: eyes which received three initial loading doses of intravitreal anti-VEGF agents at monthly intervals followed by additional PRN treatment; treatment-naïve eye with no prior treatment for nAMD; follow-up duration of at least 24 months after the first dose of intravitreal anti-VEGF injection; and availability of 3 or more OCT central subfield thickness (CST) values during the follow-up period. A treatment-agnostic approach was adapted for the study to include patients treated with any of the three available anti-VEGF agents (bevacizumab [Roche, Basel, Switzerland]; ranibizumab [Novartis, Basel, Switzerland]; and aflibercept [Bayer, Leverkusen, Germany]) because all three intravitreal anti-VEGF agents result in significant visual gain after treatment, as demonstrated in previous studies [17]. The study was conducted in accordance with the Declaration of Helsinki.

### 2.2. Optical Coherence Tomography Imaging and Measurements

Spectral-domain OCT scans were obtained using with the Cirrus HD-OCT 4000 (Carl Zeiss Meditec, Dublin, CA, USA). The “Macular Cube 512 × 128” scan protocol was used to obtain the OCT images of the macula, covering a retinal area of 6.0 × 6.0 mm. The OCT CST value of the individual OCT B-scan was obtained using the automated software and is defined as the distance between the middle of the retinal pigment epithelium and the internal limiting membrane at the central 1 mm subfield.

### 2.3. Statistical Analysis

Data were entered into computer spreadsheet software (Microsoft Excel for Mac version 16.52, Microsoft Corp, Redmond, WA, USA) and statistical analyses were carried out using a statistical module (StatPlus:mac Pro version 5.9.80, AnalystSoft Inc., Walnut, CA, USA) running within the spreadsheet software. OCT CST variability values were calculated from the standard deviation (SD) of all the available OCT CST values of the eye at each follow-up visit after the 3 loading doses of intravitreal anti-VEGF injection from month 3 to month 24. Eyes were then categorized into four quartiles of OCT CST variability, ranging from the group with the lowest variability values to the group with the highest variability values. Correlation analysis between the OCT CST variability values and the best-corrected visual acuity (BCVA) at 2 years was performed using the non-parametric Spearman’s rank test. The mean change in BCVA and the mean number of intravitreal anti-VEGF injections were compared between the 4 OCT CST variability quartile groups using one-way ANOVA tests. A *p*-value of ≤0.05 was considered as statistically significant.

## 3. Results

### 3.1. Baseline Patient Demographics

A total of 64 eyes of 62 patients were included in the study. The mean ± SD age of the patients at baseline was 75.3 ± 9.4 years (range, 50 to 91 years). All patients were of Chinese ethnicity and there were 38 (61.3%) males and 24 (38.7%) females. At baseline, the mean ± SD logMAR BCVA was 0.59 ± 0.39 (range, 0.0 to 1.3) and the mean ± SD OCT CST was 364 ± 113 µm (range, 210 to 665 µm).

### 3.2. Correlation of OCT CST Variability Values and BCVA at 2 Years

**Figure 1:**
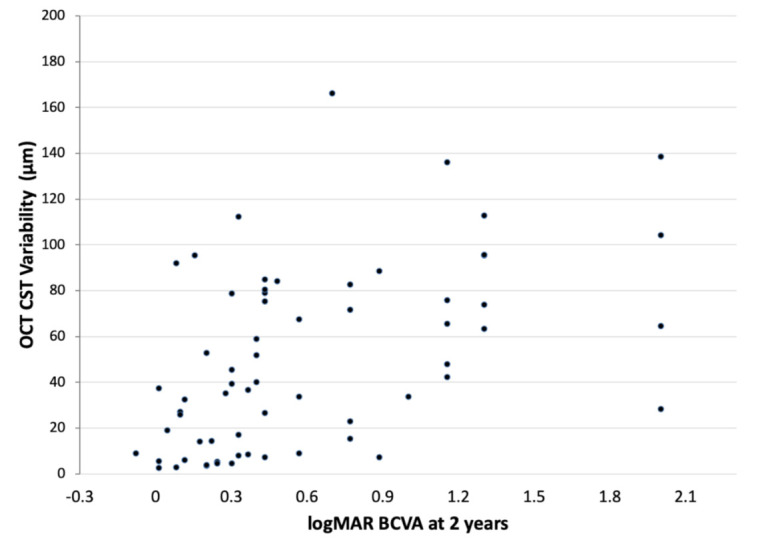
Correlation analysis between OCT CST variability and logMAR BCVA at 2 years.

The correlation between OCT CST variability values and the logMAR BCVA at 2 years is shown in Figure 1. There was a significant correlation between OCT CST variability values and the logMAR BCVA at 2 years (Spearman’s ρ = 0.54, *p* < 0.0001). This indicates that eyes which had less variability in OCT CST values during the 2-year treatment period had better visual acuity at 2 years.

### 3.3. Correlation of OCT CST Variability Quartiles and Change in BCVA at 2 Years

The OCT CST variability values for the four quartile groups are listed in Table 1. The lowest quartile (quartile 1) group had OCT CST variability values of <14.4 µm, whereas the highest quartile group (quartile 4) had OCT CST variability values of ≥78.7 µm.

**Figure 2:**
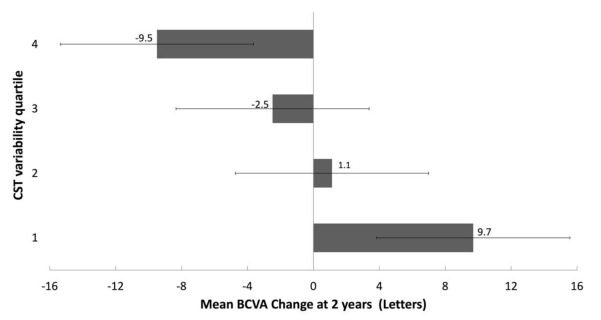
Mean BCVA change at 2 years for the CST variability quartile groups (error bars represent the 95% confidence intervals).

The mean BCVA changes at 2 years for the four OCT CST variability quartile groups are displayed in Figure 2. There was a significant difference in the mean BCVA change between the four OCT CST variability groups (*p* = 0.018). The lowest two quartiles of CST variability had BCVA gains of 9.7 letters (quartile 1) and 1.1 letters (quartile 2), whereas the highest two quartile groups of OCT CST variability had BCVA losses of 2.5 letters (quartile 3) and 9.5 letters (quartile 4).

### 3.4. Anti-VEGF Injections and OCT CST Variability Quartiles

Various intravitreal anti-VEGF agents were used in the study, including aflibercept alone in 22 (34.3%) eyes, bevacizumab alone in 17 (26.6%) eyes, ranibizumab alone in 8 (12.5%) eyes, and a combination of the two agents in 17 (26.6%) eyes. The median number of intravitreal anti-VEGF injections during the 24 months was 7 (interquartile range [IQR], 4 to 10). The median number of injections in each OCT CST variability quartile group was 3 (IQR, 3 to 6.5) for quartile 1, 7.5 (IQR, 5.5 to 11.5) for quartile 2, 7.5 (IQR, 5.5 to 9.5) for quartile 3 and 7 (IQR, 3 to 10) for quartile 4. No significant difference in the number of injections was found between the four quartile groups (one-way ANOVA, *p* = 0.23).

## 4. Discussion

In this study, we utilized a personalized treatment approach of a PRN anti-VEGF therapy dosing regimen for treating nAMD patients in order to minimize the treatment burden in terms of the number of injections and drug costs associated with anti-VEGF therapy. In many Asian countries, anti-VEGF therapy is frequently self-financed or is only partially subsided, with a cap to the maximum number of anti-VEGF injections that can be reimbursed [19,20]. Therefore, patients often choose to receive a reactive PRN dosing regimen rather than more proactive treatment approaches, such as fixed dosing or treat-and-extend regimens. It is well known that the visual acuity of a substantial proportion of patients with nAMD could still deteriorate to before-treatment levels despite receiving regular anti-VEGF therapy, especially in the long term [21]. Our study demonstrated that eyes with greater variation in OCT retinal thickness during intravitreal anti-VEGF treatment for nAMD had worse visual outcomes in terms of both final visual acuity and mean change in BCVA at 2 years compared with eyes which had smaller variation in retinal thickness. Our findings based on a real-world clinical setting using a personalized PRN treatment regimen are similar to those observed in the CATT and IVAN studies, which used protocol-driven treatment regimens in clinical trial settings [17]. With the use of an artificial intelligence algorithm to analyze the volumes of macular fluid compartments, Chakravarthy et al. also demonstrated that greater variations in the volume of retinal fluids were associated with worse visual acuity outcomes at 2 years [18].

Several reasons have been postulated to account for the worse visual outcomes in eyes which had greater variation in retinal thickness during anti-VEGF therapy for nAMD. A possible cause might be related to the increased development of fibrosis and macular atrophy [17]. Evans et al. found that the risks of developing fibrosis and macular atrophy increased with greater variation in OCT retinal thickness in eyes receiving anti-VEGF therapy for nAMD, with the highest quartile of retinal thickness variability having odds ratios of 1.95 and 2.10 in developing fibrosis and geographic atrophy, respectively [17]. Similar associations were observed regardless of whether the eye was allocated to the continuous or to the discontinuous PRN treatment groups. Another possible reason for the association between greater OCT variability and worse visual outcome might be due to possible undertreatment, because real-world studies have shown that better visual acuity outcomes might be associated with a higher number of anti-VEGF injections [22,23]. However, as observed in our study, the number of injections was similar between the four OCT CST variability quartile groups. Patients in the lowest two OCT CST variability quartiles had visual acuity gains which could be achieved with around three to four intravitreal anti-VEGF injections per year, and were similar to the highest two quartile groups. In the CATT and IVAN studies, a higher number of anti-VEGF injections was found to be associated with an increased likelihood of having greater OCT retinal thickness variability [17]. Therefore, the association between greater retinal thickness variability and worse visual acuity outcome is likely to be independent of the degree of anti-VEGF treatment.

The findings in our study suggest that when treating patients with nAMD, clinicians should aim to adopt treatment strategies which can minimize variation in OCT retinal thickness. This may be achieved by adopting a more objective individualized treat-and-extend treatment approach using OCT and visual acuity findings to increase the chance of maintaining a dry macula. A form of automated modified treat-and-extend protocol is being evaluated in the faricimab personalized treatment interval arm in the LUCERNE and TENAYA studies for nAMD [24,25]. Another potential option for minimizing the variation in retinal thickness is to utilize newer therapeutic agents which have stronger ability to resolve macular fluids or have increased treatment durability [24,25,26]. For example, brolucizumab has been shown to result in a significantly greater proportion of eyes with fluid resolution compared with aflibercept; thus, it might be useful in achieving less fluid fluctuation over the course of anti-VEGF therapy [27].

The main limitations of our current study include the retrospective nature of the study and the small number of eyes included. Due to the retrospective nature of the study, sample size and power calculations were not performed. We also only performed a qualitative assessment of the OCT findings based only on CST values; other measures such as volumes of various macular fluid compartments, fibrosis and macular atrophy were not evaluated. Nonetheless, our findings in the real-world clinical setting showed that greater variation in OCT retinal thickness following loading doses of anti-VEGF therapy for nAMD is associated with worse visual acuity outcomes at 2 years. Further studies to determine the optimal treatment regimen which can reduce or minimize retinal thickness variation to improve the treatment outcomes are warranted.

## Figures and Tables

**Table 1:** OCT CST variability values of the 4 quartile groups during the study period.

Quartile 1 (Least Variability)	Quartile 2	Quartile 3	Quartile 4 (Greatest Variability)
<14.4 µm	14.4–39.4 µm	39.4–78.7 µm	≥78.7 µm

## Data Availability

The data presented in this study are available on request from the corresponding author. The data are not publicly available due to privacy.

## References

[1] Flaxman S.R., Bourne R., Resnikoff S., Ackland P., Braithwaite T., Cicinelli M.V., Das A., Jonas J.B., Keeffe J., Kempen J.H. (2017). Global causes of blindness and distance vision impairment 1990–2020: A systematic review and meta-analysis. Lancet Glob. Health.

[2] Adamis A.P., Brittain C.J., Dandekar A., Hopkins J.J. (2020). Building on the success of anti-vascular endothelial growth factor therapy: A vision for the next decade. Eye.

[3] Sloan F.A., Hanrahan B.W. (2014). The effects of technological advances on outcomes for elderly persons with exudative age-related macular degeneration. JAMA Ophthalmol..

[4] Finger R.P., Daien V., Eldem B.M., Talks J.S., Korobelnik J.F., Mitchell P., Sakamoto T., Wong T.Y., Pantiri K., Carrasco J. (2020). Anti-vascular endothelial growth factor in neovascular age-related macular degeneration-a systematic review of the impact of anti-VEGF on patient outcomes and healthcare systems. BMC Ophthalmol..

[5] Heath Jeffery R.C., Mukhtar S.A., Lopez D., Preen D.B., McAllister I.L., Mackey D.A., Morlet N., Morgan W.H., Chen F.K. (2021). Incidence of newly registered blindness from age-related macular degeneration in Australia over a 21-Year Period: 1996–2016. Asia Pac. J. Ophthalmol..

[6] Mantel I. (2015). Optimizing the anti-VEGF treatment strategy for neovascular age-related macular degeneration: From clinical trials to real-life requirements. Transl. Vis. Sci. Technol..

[7] García-Layana A., Figueroa M.S., Arias L., Araiz J., Ruiz-Moreno J.M., García-Arumí J., Gómez-Ulla F., López-Gálvez M.I., Cabrera-López F., García-Campos J.M. (2015). Individualized therapy with ranibizumab in wet age-related macular degeneration. J. Ophthalmol..

[8] Garweg J.G., Niderprim S.A., Russ H.M., Pfister I.B. (2017). Comparison of strategies of treatment with ranibizumab in newly-diagnosed cases of neovascular age-related macular degeneration. J. Ocul. Pharmacol. Ther..

[9] Hatz K., Prünte C. (2017). Treat and extend versus pro re nata regimens of ranibizumab in neovascular age-related macular degeneration: A comparative 12 month study. Acta Opphthalmol..

[10] Chaikitmongkol V., Sagong M., Lai T.Y.Y., Tan G.S.W., Fariza N., Ohji M., Mitchell P., Yang C.H., Ruamviboonsuk P., Wong I. (2021). Treat-and-extend regimens for the management of neovascular age-related macular degeneration and polypoidal choroidal vasculopathy: Consensus and recommendations from the Asia-Pacific Vitreo-Retina Society. Asia Pac. J. Ophthalmol..

[11] Okada M., Kandasamy R., Chong E.W., McGuiness M., Guymer R.H. (2018). The treat-and-extend injection regimen versus alternate dosing strategies in age-related macular degeneration: A systematic review and meta-analysis. Am. J. Ophthalmol..

[12] Ross A.H., Downey L., Devonport H., Gale R.P., Kotagiri A., Mahmood S., Mehta H., Narendran N., Patel P.J., Parmar N. (2020). Recommendations by a UK expert panel on an aflibercept treat-and-extend pathway for the treatment of neovascular age-related macular degeneration. Eye.

[13] Spaide R.F. (2021). Antivascular endothelial growth factor dosing and expected acuity outcome at 1 year. Retina.

[14] Phadikar P., Saxena S., Ruia S., Lai T.Y., Meyer C.H., Eliott D. (2017). The potential of spectral domain optical coherence tomography imaging based retinal biomarkers. Int. J. Retin. Vitr..

[15] Zhang X., Lai T.Y.Y. (2018). Baseline predictors of visual acuity outcome in patients with wet age-related macular degeneration. BioMed Res. Int..

[16] Nguyen V., Puzo M., Sanchez-Monroy J., Gabrielle P.H., Garcher C.C., Baudin F., Wolff B., Castelnovo L., Michel G., O’Toole L. (2021). Association between anatomical and clinical outcomes of neovascular age-related macular degeneration treated with antivascular endothelial growth factor. Retina.

[17] Evans R.N., Reeves B.C., Maguire M.G., Martin D.F., Muldrew A., Peto T., Rogers C., Chakravarthy U. (2020). Associations of variation in retinal thickness with visual acuity and anatomic outcomes in eyes with neovascular age-related macular degeneration lesions treated with anti-vascular endothelial growth factor agents. JAMA Ophthalmol..

[18] Kaiser P.K., Wykoff C.C., Singh R.P., Khanani A.M., Do D.V., Patel H., Patel N. (2021). Retinal fluid and thickness as measures of disease activity in neovascular age-related macular degeneration. Retina.

[19] Eldem B., Lai T.Y.Y., Ngah N.F., Vote B., Yu H.G., Fabre A., Backer A., Clunas N.J. (2018). An analysis of ranibizumab treatment and visual outcomes in real-world settings: The UNCOVER study. Graefes Arch. Clin. Exp. Ophthalmol..

[20] Lai T.Y.Y., Cheung C.M.G., Mieler W.F. (2017). Ophthalmic application of anti-VEGF therapy. Asia Pac. J. Ophthalmol..

[21] Chandra S., Arpa C., Menon D., Khalid H., Hamilton R., Nicholson L., Pal B., Fasolo S., Hykin P., Keane P.A. (2020). Ten-year outcomes of antivascular endothelial growth factor therapy in neovascular age-related macular degeneration. Eye.

[22] Ciulla T.A., Hussain R.M., Pollack J.S., Williams D.F. (2020). Visual acuity outcomes and anti-vascular endothelial growth factor therapy intensity in neovascular age-related macular degeneration patients: A real-world analysis of 49 485 Eyes. Ophthalmol. Retin..

[23] Chandra S., Rasheed R., Menon D., Patrao N., Lamin A., Gurudas S., Balaskas K., Patel P.J., Ali N., Sivaprasad S. (2021). Impact of injection frequency on 5-year real-world visual acuity outcomes of aflibercept therapy for neovascular age-related macular degeneration. Eye.

[24] Nicolò M., Ferro Desideri L., Vagge A., Traverso C.E. (2021). Faricimab: An investigational agent targeting the Tie-2/angiopoietin pathway and VEGF-A for the treatment of retinal diseases. Expert Opin. Investig. Drugs.

[25] Khanani A.M., Russell M.W., Aziz A.A., Danzig C.J., Weng C.Y., Eichenbaum D.A., Singh R.P. (2021). Angiopoietins as potential targets in management of retinal disease. Clin. Ophthalmol..

[26] Iglicki M., González D.P., Loewenstein A., Zur D. (2021). Longer-acting treatments for neovascular age-related macular degeneration-present and future. Eye.

[27] Dugel P.U., Singh R.P., Koh A., Ogura Y., Weissgerber G., Gedif K., Jaffe G.J., Tadayoni R., Schmidt-Erfurth U., Holz F.G. (2021). HAWK and HARRIER: Ninety-Six-Week Outcomes from the Phase 3 Trials of Brolucizumab for Neovascular Age-Related Macular Degeneration. Ophthalmology.

